# Improving time to palliative care review with predictive modeling in an inpatient adult population: study protocol for a stepped-wedge, pragmatic randomized controlled trial

**DOI:** 10.1186/s13063-021-05546-5

**Published:** 2021-09-16

**Authors:** Patrick M. Wilson, Lindsey M. Philpot, Priya Ramar, Curtis B. Storlie, Jacob Strand, Alisha A. Morgan, Shusaku W. Asai, Jon O. Ebbert, Vitaly D. Herasevich, Jalal Soleimani, Brian W. Pickering

**Affiliations:** 1 Kern Center for the Science of Health Care Delivery, Mayo Clinic, Rochester, MN USA; 2 Department of Quantitative Health Sciences, Mayo Clinic, MN 55905 Rochester, USA; 3 Department of Medicine, Mayo Clinic, Rochester, MN 55905 USA; 4 Center for Palliative Medicine, Mayo Clinic, Rochester, MN USA; 5 Department of Anesthesiology, Mayo Clinic, Rochester, MN 55905 USA

**Keywords:** Palliative care, Electronic medical record, Artificial intelligence, AI, Pragmatic clinical trials, Stepped wedge trials

## Abstract

**Background:**

Palliative care is a medical specialty centered on improving the quality of life (QOL) of patients with complex or life-threatening illnesses. The need for palliative care is increasing and with that the rigorous testing of triage tools that can be used quickly and reliably to identify patients that may benefit from palliative care.

**Methods:**

To that aim, we will conduct a two-armed stepped-wedge cluster randomized trial rolled out to two inpatient hospitals to evaluate whether a machine learning algorithm accurately identifies patients who may benefit from a comprehensive review by a palliative care specialist and decreases time to receiving a palliative care consult in hospital. This is a single-center study which will be conducted from August 2019 to November 2020 at Saint Mary’s Hospital & Methodist Hospital both within Mayo Clinic Rochester in Minnesota. Clusters will be nursing units which will be chosen to be a mix of complex patients from Cardiology, Critical Care, and Oncology and had previously established relationships with palliative medicine. The stepped wedge design will have 12 units allocated to a design matrix of 5 treatment wedges. Each wedge will last 75 days resulting in a study period of 12 months of recruitment unless otherwise specified. Data will be analyzed with Bayesian hierarchical models with credible intervals denoting statistical significance.

**Discussion:**

This intervention offers a pragmatic approach to delivering specialty palliative care to hospital patients in need using machine learning, thereby leading to high value care and improved outcomes. It is not enough for AI to be utilized by simply publishing research showing predictive performance; clinical trials demonstrating better outcomes are critically needed. Furthermore, the deployment of an AI algorithm is a complex process that requires multiple teams with varying skill sets. To evaluate a deployed AI, a pragmatic clinical trial can accommodate the difficulties of clinical practice while retaining scientific rigor.

**Trial registration:**

ClinicalTrials.gov NCT03976297. Registered on 6 June 2019, prior to trial start.

**Supplementary Information:**

The online version contains supplementary material available at 10.1186/s13063-021-05546-5.

## Background

Palliative care is a medical specialty centered on improving the quality of life (QOL) of patients with complex or life-threatening illnesses through the prevention and management of pain and other problems, be it physical, psychosocial, and/or spiritual [1].

As patient populations in western countries are aging and becoming more complex, often requiring care from multiple specialties, there is a growing recognition of the need for specialty palliative care teams to engage with patients earlier in their disease course. A consequence of this increased demand is exemplified by the growing mismatch between clinical care and patient preferences at the end-of-life. For example, research has demonstrated that most people prefer to die at home despite the majority dying outside of the home (nursing home or hospital) [2, 3]. Palliative care specialists excel in goals of care and patient preference discussions. Unfortunately, palliative care expertise is often an underutilized resource, and the current model of care and incentives may relegate palliative care as a “last resort” after all attempts at cure have been exhausted [4]. This delay can lead to suboptimal symptom management for pain, lower quality of life, and even reduced survival [5–8]. In addition to these unrealized benefits, palliative care has been shown to be inversely associated with aggressive medical interventions and emergency room (ER) visits, hospital and intensive care unit (ICU) admission, and death in the last weeks of life, all of which are indicative of low value care [9–11]. Demand for appropriate palliative care will and should increase, and with that, policy initiatives and referral triage tools that lead to timely high-quality palliative care services will need to be implemented [12]. Triage tools can be used quickly and reliably to identify patients that may benefit from palliative care, thereby leading to high value care and improved outcomes.

### Risk scores

The development of risk stratification and triage tools for palliative care services has an extensive history with early methods such as The Palliative Prognostic Score (PaP) [13, 14]. Although a complete enumeration of scores in this space is out of scope, there are three aspects of palliative care scoring that are important to mention. First, many palliative care tools often use proxies for palliative care need. For example, mortality tends to be the most common proxy usually in the form of the surprise question—“Would I be surprised if this patient died in the next 12 months?” Although straightforward, mortality as a proxy is substandard, because many patients who are at risk of mortality do not necessarily need palliative care and many patients can benefit from palliative care interaction, even if their risk of short-term mortality is low. Other scores have modeled proxies such as use of hospice services but still limit care to end of life and not the entire spectrum of palliative care needs [15, 16]. Second, palliative care tools often have a targeted timeframe or specified population. For instance, early palliative care scores were focused on patients with cancer, whether late stage or early stage [17]. Subsequently, scores have been developed specifically for inpatient, either general floor or ICU units or community based [18–20]. Although these scores have greater utility, they limit the ability to transfer to other populations which may limit legitimate palliative care need. Third, the complexity of a score is often an important consideration. Earlier models have utilized scores that were simple and easy to calculate and could be done through clinical examination. Recently with the advent of artificial intelligence (AI) and near universal adoption of electronic medical records (EMR), scores are now being developed that utilizes large and complex types of routinely collected data for predictions. Avati et al. developed a deep learning algorithm on EMR data utilizing a model with 13,654 features predicting mortality, and Jung et al. developed an outpatient mortality prediction algorithm utilizing 1880 features and using gradient boosting machines (GBMs) [20, 21].

### Development of integrated palliative risk score algorithm

Informed by these considerations, a machine learning algorithm trained on EMR data and fully integrated into an IT solution focusing on the identification of patients who may benefit from early palliative care review was developed on inpatients from a large academic medical center. The solution, known as Control Tower, pulls disparate data sources centered on a machine learning algorithm which predicts the need for palliative care in hospital. The algorithm along with other key patient indicators was integrated into a graphical user interface (GUI) which allows a human operator, known as the Control Tower Operator (CTO) to review the algorithm predictions and subsequently record the operator’s assessment.

### Evaluation of AI

Despite the large number of published risk scores in the literature for palliative care needs, to the best of our knowledge, there has been a lack of research assessing wide scale *evaluation and implementation* of palliative risk scores into clinical practice. Often scores are tested based solely on predictive performance with validation consisting of clinical review. As hypothesized by Spiegelhalter, it is not enough for algorithms to be trusted by simply publishing research showing predictive performance (phase I) or clinical assessment (phase II); two additional phases are needed. First, there should be field testing with clinical trials demonstrating an impact on clinical outcomes (phase III) as well as an infrastructure to ensure prospective monitoring during routine use [22]. There have been some encouraging and notable exceptions nonetheless; in Courtright et al., the authors published an EHR algorithm for palliative care with a pre-post evaluation on frequencies of consults, advance care planning (ACP) documentation, home palliative care and hospice referrals, code status changes, and length of stay [23].

### Aims of the study

The objective of this study is to evaluate whether the Control Tower solution accurately identifies patients who may benefit from a comprehensive review by a palliative care specialist and decreases time to receiving a palliative care consult in hospital. By creating an algorithm that automatically screens and monitors patient health status during their hospitalization, the hypothesis being tested is that patients will receive *needed* palliative care earlier than under the usual course of care.

### Trial design

To accomplish the primary aim, we will use a two-armed stepped-wedge cluster randomized trial rolled out to both inpatient hospitals in Mayo Clinic, Rochester. Trial units will receive a computerized assessment of patients in the hospital identified to be at risk of needing palliative care services where control units will receive the usual source of care.

## Methods/design

### Study setting

This is a single-center study which will be conducted from August 2019 to November 2020 at Saint Mary’s Hospital & Methodist Hospital, Mayo Clinic, Rochester, Minnesota. The nursing units chosen for the study have a mix of complex patients from Cardiology, Critical Care, and Oncology and had previously established relationships with palliative medicine.

### Eligibility criteria

The recruitment and enrollment is broad and is designed to mimic the actual use of the Control Tower in practice. To be included in the trial, patients will need to be admitted to either inpatient facility during the study period. Patients will need to have a risk score of at least 7 (out of 100) from the algorithm. Patients will be excluded from review if they under the age of 18, previously seen by palliative care during the current hospital encounter, currently enrolled in hospice, or currently followed by a palliative care team. In addition, patients with an expected discharge in the next 24 h and patients who do not provide research authorization to review their medical records for general research studies in accordance with Minnesota Statute 144.335 will be excluded from the study.

### Intervention

**Fig. 1:**
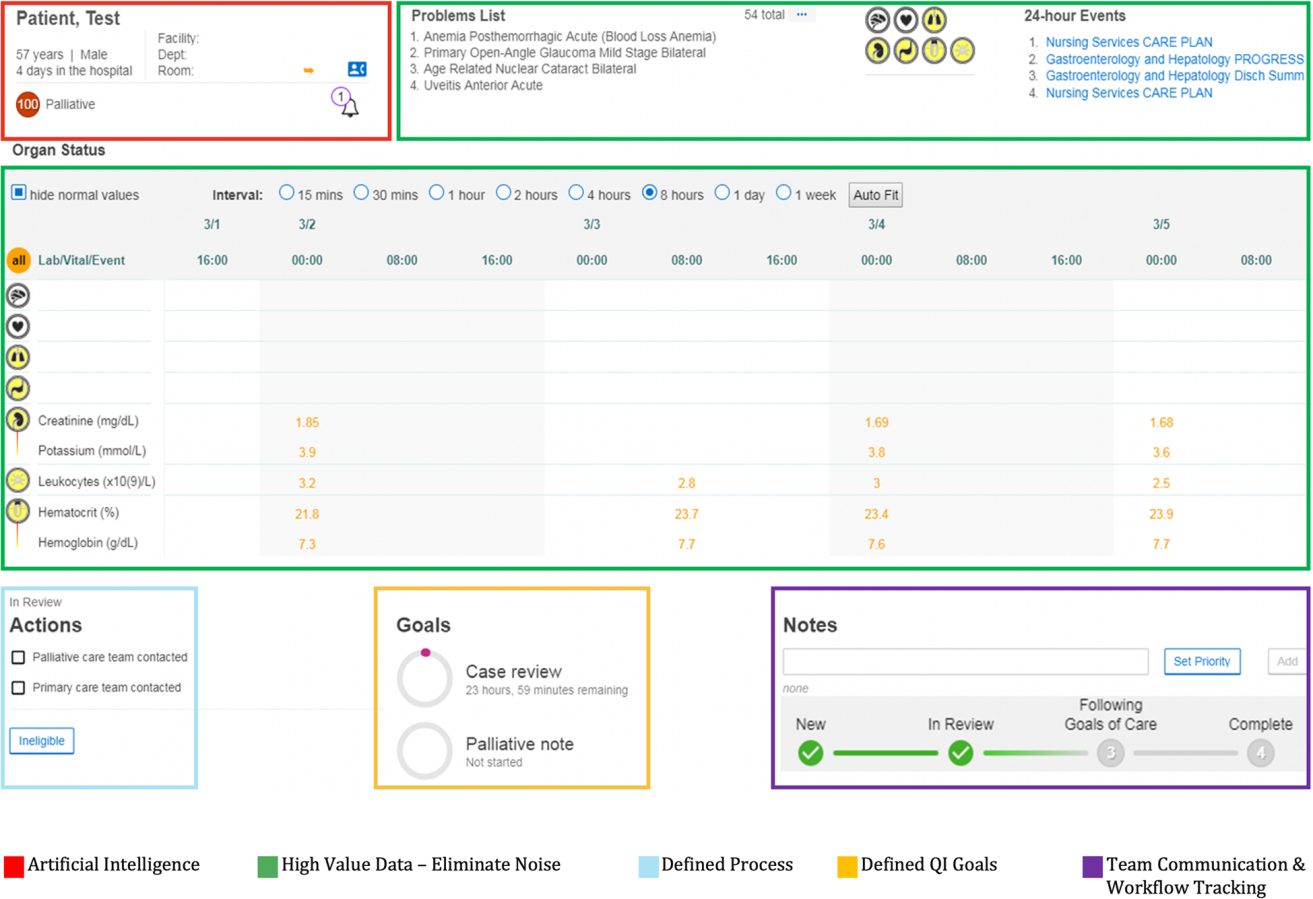
Screenshot of the Control Tower user interface

A full description of the Control Tower interface can be learned through Murphee et al. [24]. Briefly, the Control Tower is a workstation and software tool that extracts medical data, processes the prediction algorithm, and presents the results through an ordered patient list. Currently, the algorithm is running on all inpatients in both study hospitals in an automated monitored process. A screenshot of the interface can be seen in Fig. 1. In addition to the algorithmic score, additional data on comorbidities, laboratory values, and hospital events are available and presented to give the score context. Patients receive scores from the Control Tower (0–100; higher scores indicate increased need) for palliative care and are subsequently ranked from highest to lowest need with each score colored into tertiles: red (7 or greater), yellow (less than 7, greater than or equal to 4), and white (less than 4). Patients with previous palliative care in their hospital stay have their scores labeled green.

The intervention will include a CTO who will interact with the inpatient palliative care consult service at both study sites. The CTO will monitor the Control Tower during weekday normal business hours (Monday through Friday; 8 am–5 pm) and select, once a day, a cohort of patients with the highest need who may benefit from palliative care review. The operator will assess for any additional exclusion criteria in developing the final list. After all screening is finished, the CTO will select the top 12 patients to be sent to palliative care in a file through email. The number 12 was agreed upon to match the expected capacity of the palliative care team and to maintain regularity throughout the trial. The file will consist of the patient identifying information, along with the algorithmic score indicating probability of needing palliative care, and contextual factors such as the hospital unit they are in and what factors in the model are driving the score. The palliative care team member who is on service will also assess the need for each patient through the daily report and record whether they agreed with the algorithm’s conclusion or not. For those agreed upon patients who are also in the intervention arm, the palliative care team will approach the attending clinical team to suggest a palliative care referral for the patient.

The utilization of a new tool such as the Control Tower to identify patients appropriate for palliative care can be disruptive to standard clinical workflows and processes. To help ensure proper dissemination of these new referral patterns, we engaged with a communications specialist and worked directly with area practice leaders to set up a communication plan.

Patients who are not in an intervention period will receive the standard of care. The usual source of care for both the intervention group and the control group delivered by the specialty palliative care consultation intervention will remain consistent, with a billable palliative care provider conducting a comprehensive palliative care review (symptom assessment, psychosocial-spiritual assessment, goals of care discussion and recommendations as indicated) along with involvement of an interdisciplinary team (evaluation with specialty palliative care nurses, social worker, chaplain). This is feasible given we can easily control the communication between the palliative care team and the attending teams to prevent any contamination between clusters. We calibrated the prediction model and the Control Tower review to match the average capacity of the palliative care service, knowing that that they will still receive palliative care consults through the traditional pathway of the attending care team consulting Palliative Medicine directly. This additional measure increases the likelihood the control group gets the usual source of care.

### Outcomes

For all study outcomes, data will be collected through either the electronic medical record or administrative billing system at trial completion and unless otherwise noted each outcome will be determined during the patient’s inpatient stay. The primary outcome will be time to palliative care as measured by the electronic record of a consult by Palliative Medicine. Data will go through quality checking quarterly, at each step during the burn-in period, and before finalization.

The secondary outcomes are as follows:

1. The number of inpatient palliative care consults—measured by the number of palliative care consults in the inpatient units of interest.
2. Time to palliative care per unit—measured as time in hours from alert to the electronic record of consult by the palliative care team in the inpatient setting for each of the 12 nursing units.
3. Transition time to hospice-designated bed—for all patients with Medicare insurance the time from alert until transferred to a hospice-designated bed for an inpatient encounter.
4. Time to hospice designation—measured as time in hours from alert to the electronic record of consult by the hospice care team for an inpatient encounter.
5. Emergency Department visit within 30 days of discharge—measured by the number of study participants who upon discharge from the inpatient setting are readmitted to the Emergency Department at any Mayo Clinic facility within 30 days (excluding inpatient readmissions through the Emergency Department).
6. Hospitalization or readmission within 30 days of discharge—measured by the number of study participants who upon discharge from the inpatient setting are readmitted to an inpatient unit at any Mayo Clinic facility within 30 days (excluding transfers and planned readmits).
7. ICU transfers—measured by the number of study participants who transferred to an ICU during their inpatient stay.
8. Ratio of inpatient hospice death to non-hospice hospital deaths—measured by the number of deaths of study participants in hospice designated beds by the number of deaths in non-hospice beds.
9. Rate of discharge to external hospice—measured by the number of participants whose electronic health record indicates discharge to external hospice.
10. Inpatient length of stay—measured as time from admission to discharge from hospital for all study participants.

### Participant timeline

**Fig. 2:**
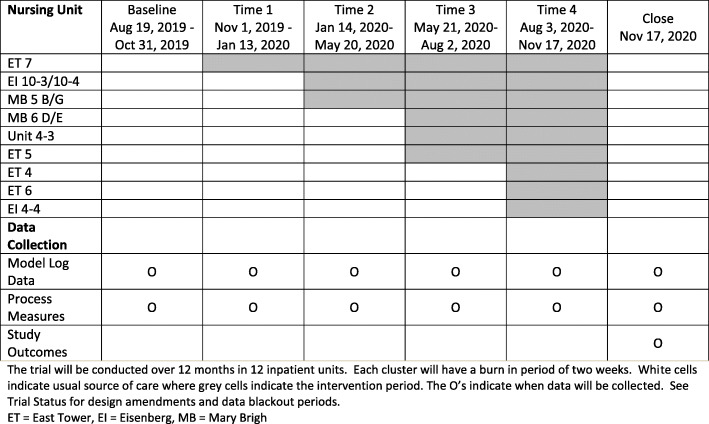
Stepped wedge design

In the stepped-wedge design clusters, in this case floor units, cross over randomly (computer generated) from the control or standard of care condition to the intervention condition in a staggered fashion. The stepped wedge design will have 12 units allocated to a design matrix of 5 treatment wedges. Each wedge will last 75 days resulting in a study period of 12 months unless otherwise specified. The first step will be a baseline period in which no intervention is administered, where in the last step all clusters will be treated (Fig. 2). At the start of each wedge, there will be 2 weeks of burn-in to allow the clinical team to integrate the intervention with their workflow. Due to the pragmatic nature of the design, we are unable to blind providers to whether they were in the intervention unit or control unit. Patients will receive the same type of palliative medicine consult as they would normally receive in hospital, with the only difference being in how they were alerted. There was no additional data collected from patients and no follow up visits (i.e., all care will be done through the patient’s hospitalization).

### Data analysis plan

Summarized patient data will be characterized by age, sex, and baseline covariates entered into the machine learning algorithm (HCC diagnoses codes, previous utilization and lab measurements). All patients will be analyzed on an intention to treat status; this principle will be extended to the cluster status in the event of transfers between intervention and control units.

**Fig. 3:**
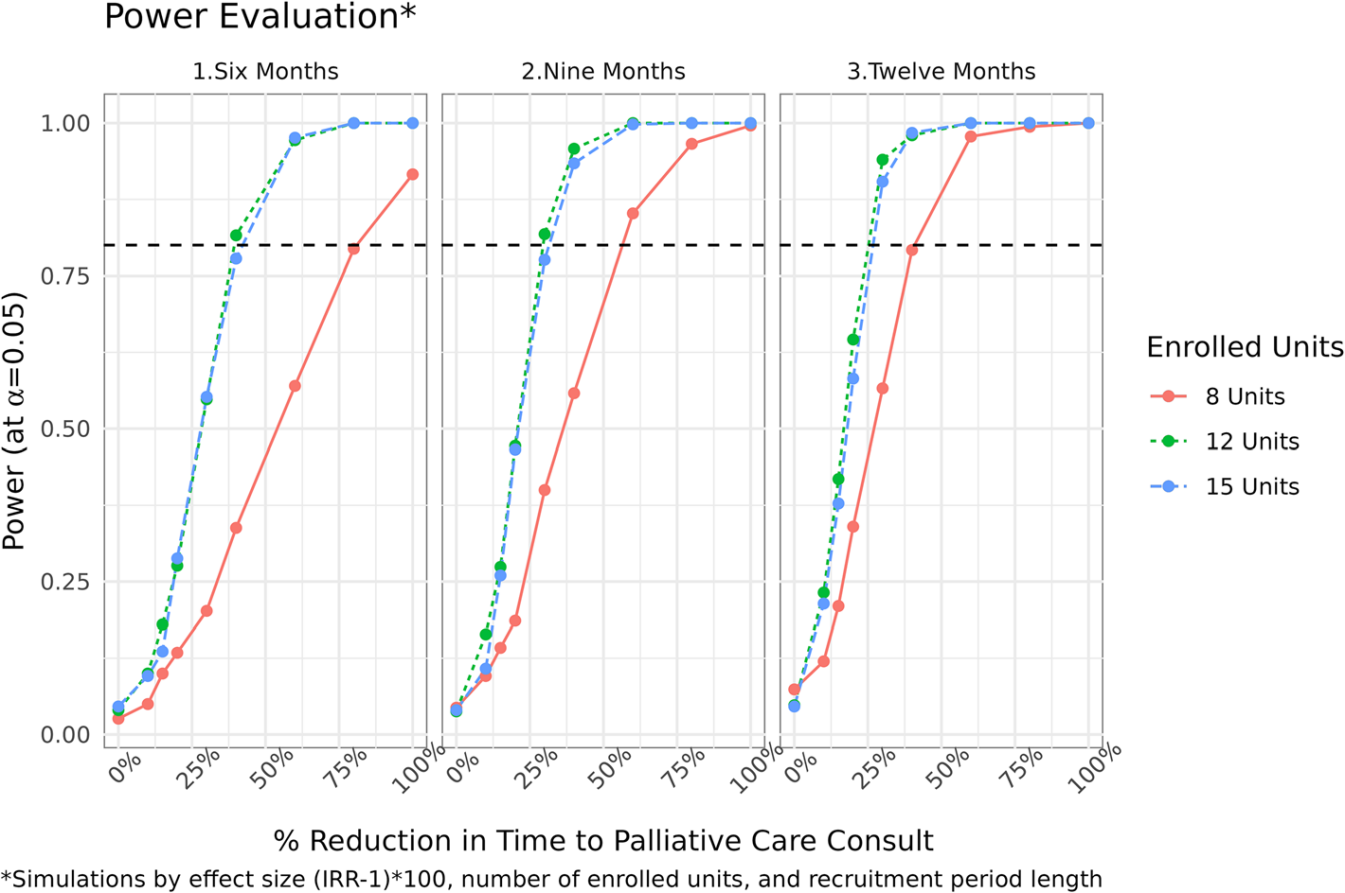
Stepped wedge design power curves

For all study outcomes, Bayesian estimation to account for design features in the stepped design will be used. Specially, time-to-event modeling to assess the effects of the intervention will be used to model time to palliative care and other time to event or count outcomes. The chosen model consists of a hierarchical regression treating the time-to-event as a heterogeneous Poisson process, allowing for adjustment to the event rate due to secular time effects and unit clustering. Our model is as follows: let Yijt indicate whether the ith eligible patient at the jth nursing unit during time t (t = 0,…,4) receives a palliative care consult during period t. Then, according to the stepped wedge designYijt ~ Poisson(λijt)log⁡(λijt) = μ + αj[i] + β1X[i] + δt[i] + 1*log(offset[i]) (1)where μ is the overall mean, and αj is a random effect for nursing unit (cluster) j, j=1, 2, …12, αj[i] ~ N(0,τ2). δt[i] is the multivariate autoregressive process of order 1, AR (1), with Gaussian noise to adjust for the secular trend, X[i] indicates the intervention at the jth clinical practice at time t. β1 capture the main effect for the intervention, and the offset is the patient’s time spent in the j nursing unit at time t (1). Statistical tests will be based on 95% credible intervals and effect estimates will be Incident rate ratios (IRR). For secondary binary outcomes, logistic regression will be used with the same design features. Stepped-wedge cluster randomization trials typically have more statistical power than other cluster randomized designs when clusters are correlated, because each cluster is able to serve as its own control. Because of the complex nature of the design, there was no suitable closed formed solution for sample size thus requiring the estimation of statistical power using Monte Carlo simulation [25]. Our model for the simulation consisted of the same hierarchical Poisson regression model (1). In Monte Carlo simulation, you design a model and repeatedly draw samples under a set of hypothetical sample sizes, treatment effects, and fixed nuisance parameters. Then, for each sample, you test your hypothesis and collect the number of positive results; this is your estimated power. To estimate reasonable parameters for the nursing unit mean and time trends for receiving palliative care consultation, we collected pilot data for all Mayo Rochester inpatient admissions in 2017 with palliative care consult status. With estimates of the nursing unit mean and secular trend, we have at least 80% power for the 12-month recruitment timeframe with 12 nursing units and an expected positive predictive value of 50% to detect incident rate ratios (IRRs) of 1.25 or greater. See Fig. 3 for the power curves; we tested various scenarios with varying number of nursing units and recruitment lengths. We choose the 12-month recruitment time frame and 12 nursing units because it was a good compromise between the power of the test (assuring we could detect a reasonable effect) and implementation (Palliative Medicine wanted to make sure they could set up a well-defined and accepted process on each of the selected intervention units to ensure intervention fidelity). Our final simulated dataset consisted of 2294 eligible encounters. The average cluster size was 143.4 encounters with a q1 and q3 of 49.5 and 200.5, respectively. The palliative care rate with no treatment was 18.4% with the highest cluster being 20.8% and the lowest cluster being 9.2%. There was a moderate to strong correlation in the day-to-day time effect of the AR(1) parameter (*r* = 0.67).

### Data management

All data for all study outcomes, model covariates, and process measures will be collected through three principal means:

1. All input predictors and model predictions from the machine learning model are logged every time the algorithm is called and our stored in a study database.
2. Study outcomes will be collected through electronically pulling administrative billing data or data from the health systems EHR.
3. Process measures (number of Palliative Medicine accepts and reasons for rejection) will be collected through the daily logs transferred between the CTO and palliative care team.

For each variable pulled electronically, we will do a validation study to make sure that it is measuring the appropriate concept.

### Data monitoring

The proposed intervention does not exceed the threshold of minimal risk as determined by the IRB, so no data monitoring committee (DMC) will be created. Pursuant to this, there will be no interim analyses or stopping rules for ending the trial early. Risks of this study to patients are expected to not differ from those encountered during routine clinical care. Patient safety will be maintained through the clinical staff adhering to the standards of clinical care. Study logs will be audited on a quarterly basis for reporting purposes, but no statistical analyses will be done and there will be no decisions made on the data to stop or continue the trial. Study logs will be evaluated by three groups: Palliative Medicine and CTO staff, statistical analysts, and IT. Palliative Medicine and the CTO will monitor the Control Tower while using the tool to look for any errors that would impact their workflow, e.g., predictions not showing up, data elements that are clearly wrong. Study statisticians and analysts will review the study logs to make sure the fields are filled out and that the cohorts are roughly the same size week to week. IT will monitor the data pipelines to ensure that all data systems are properly working for score calculation.

### Confidentiality

Patient’s participation is only through the utilization of hospital services with no additional contact or visits needed; therefore, the hospital’s policies and procedures for maintaining patient privacy with respect to data will be in place. All patient data are securely stored behind an electronic firewall and will be stored on separate, password-protected, secure servers; only study personnel will have access to these data. For report purposes, we will use the Centers for Medicare and Medicaid Services (CMS) data protocol; all results will be reported in aggregate with no cells size smaller than 10.

### Dissemination policy

Every attempt will be made to have our work published in the literature regardless of outcome and trial summary results will be submitted to ClinicalTrials.gov following the completion of the trial. The team will follow any standard authorship requirements as specified in journals we attempt to publish in.

A checklist of recommended items to address in a clinical trial protocol according to the “Standard Protocol Items: Recommendations for Interventional Trials (SPIRIT) 2013” guidelines is also provided (see Additional file 1).

## Discussion

This study investigates the effect of a machine learning algorithm integrated into a healthcare delivery model to bring palliative care to patients sooner in the hospital.

Significant knowledge gaps exist about how risk algorithms routinely perform in practice settings due to the complex nature of the task. Building an algorithm, creating the infrastructure to deliver it in real time, and developing a healthcare model that integrates the algorithm into healthcare delivery and improves outcomes require a large interdisciplinary team. Not surprisingly, the development of such a team is often difficult to create and maintain. In the current paradigm of scientific funding and recognition, it may often be a risky decision to engage in team science vs. research which focuses on smaller scale efforts that lead to more research publications [26, 27].

Given the nature of this pragmatic clinical trial, there are several strengths and limitations that are worth noting. As this trial is imbedded into the clinical practice, the patient population is well represented with very few exclusion criteria and a waiver for both patient and provider consent. Additionally, this trial has considerably less burden; as the number and complexity of study visits, study procedures, and questionnaire burden are non-existent; and as the assessments carried out by study staff are intrinsic to the healthcare model, meaning that if implemented they would be a part of an intervention and are minimal as to not disrupt the usual source of care.

However pragmatic research is not without its limitations. Principally, the trial relies on routinely collected data and exclusively uses EHR data for study outcomes. Take our primary outcome as an instance; the choice of time to palliative care is largely a pragmatic measure due to the routine storage of specialty consults in the EHR. Although seemingly more important measures such as unnecessary utilization or quality of life could be ascertained, we opted to target a measure that had adequate power and could be routinely collected throughout the trial without too much burden to practice. In addition, the ability to validate EHR measures is limited; the ability to do small validation studies is feasible for key measures but full adjudication is not possible.

## Supplementary Information

**Additional file 1.** SPIRIT 2013 Checklist: Recommended items to address in a clinical trial protocol and related documents*.

## Data Availability

De-identified datasets will be available to qualified investigators through communication of reasonable requests with the PI after primary results manuscripts are accepted.

## Abbreviations

QOL: Quality of life
ICU: Intensive care unit
ER: Emergency room
AI: Artificial intelligence
EMR: Electronic medical record
PaP: Palliative Prognostic Score
GBM: Gradient boosting machine
GUI: Graphical user interface
CTO: Control tower operator
ACP: Advanced care planning
IRR: Incidence rate ratio
CMS: Centers for Medicare and Medicaid Services
MB: Mary Brigh Unit of Saint Mary’s Hospital
ET: East Tower of Saint Mary’s Hospital
EI: Eisenberg Unit of Methodist Hospital
